# Unhealthy Ultra-Processed Food, Diet Quality and Adherence to the Mediterranean Diet in Children and Adolescents: The DELICIOUS Project

**DOI:** 10.3390/foods14152648

**Published:** 2025-07-28

**Authors:** Francesca Giampieri, Alice Rosi, Evelyn Frias-Toral, Osama Abdelkarim, Mohamed Aly, Achraf Ammar, Raynier Zambrano-Villacres, Juancho Pons, Laura Vázquez-Araújo, Nunzia Decembrino, Alessandro Scuderi, Alice Leonardi, Lorenzo Monasta, Fernando Maniega Legarda, Ana Mata, Adrián Chacón, Pablo Busó, Giuseppe Grosso

**Affiliations:** 1 Department of Clinical Sciences, Università Politecnica delle Marche, 60131 Ancona, Italy; 2 Research Group on Food, Nutritional Biochemistry and Health, Universidad Europea del Atlántico, Isabel Torres 21, 39011 Santander, Spain; 3 Joint Laboratory on Food Science, Nutrition, and Intelligent Processing of Foods, Polytechnic University of Marche, Italy, Universidad Europea del Atlántico Spain and Jiangsu University, China; 4 International Research Center for Food Nutrition and Safety, Jiangsu University, Zhenjiang 212013, China; 5 Human Nutrition Unit, Department of Food and Drug, University of Parma, 43124 Parma, Italy; 6 School of Medicine, Universidad Católica de Santiago de Guayaquil, Av. Pdte. Carlos Julio Arosemena Tola, Guayaquil 090615, Ecuador; 7 Division of Research, Texas State University, 601 University Dr, San Marcos, TX 78666, USA; 8 Faculty of Sport Sciences, Assiut University, Assiut 71515, Egypt; 9 ESLSCA University Egypt, Giza 12676, Egypt; 10 Department of Training and Movement Science, Institute of Sport Science, Johannes Gutenberg-University Mainz, 55122 Mainz, Germany; 11 Research Laboratory, Molecular Bases of Human Pathology, LR19ES13, Faculty of Medicine, University of Sfax, Sfax 3000, Tunisia; 12 Escuela de Nutricion y Dietetica, Universidad Espíritu Santo, Samborondón 0901952, Ecuador; 13 Editorial Luis Vives (EDELVIVES), Carretera de Madrid, 50012 Zaragoza, Spain; 14 BCC Innovation, Technology Center in Gastronomy, Basque Culinary Center, 20009 Donostia-San Sebastián, Spain; 15 Basque Culinary Center, Faculty of Gastronomic Sciences, Mondragon Unibertsitatea, 20009 Donostia-San Sebastián, Spain; 16 Neonatal Intensive Care Unit, University Hospital “Policlinico-San Marco” Catania, Integrated Department for Maternal and Child’s Health Protection, 95100 Catania, Italy; 17 Department of Agriculture, Food and Environment, University of Catania, Via S. Sofia 100, 95123 Catania, Italy; 18 Department of Biomedical and Biotechnological Sciences, University of Catania, 95123 Catania, Italy; 19 Institute for Maternal and Child Health—IRCCS Burlo Garofolo, 34137 Trieste, Italy; 20 Faculty of Health Science, Universidade Internacional do Cuanza, Cuito, Bié, Angola; 21 Faculty of Health Science, Universidad de La Romana, La Romana 22000, Dominican Republic; 22 Technological Institute for Children’s Products & Leisure AIJU, 03440 Alicante, Spain; 23 Center for Human Nutrition and Mediterranean Foods (NUTREA), University of Catania, 95123 Catania, Italy

**Keywords:** ultra-processed food, Mediterranean diet, children and adolescents

## Abstract

Background: Western dietary patterns worldwide are increasingly dominated by energy-dense, nutrient-deficient industrial foods, often identified as ultra-processed foods (UPFs). Such products may have detrimental health implications, particularly if nutritionally inadequate. This study aimed to examine the intake of unhealthy UPFs among children and adolescents from five Mediterranean countries (Italy, Spain, Portugal, Egypt, and Lebanon) involved in the DELICIOUS project and to assess the association with dietary quality indicators. Methods: A survey was conducted with a sample of 2011 parents of children and adolescents aged 6 to 17 years to evaluate their dietary habits. Diet quality was assessed using the Youth Healthy Eating Index (Y-HEI), the KIDMED index to determine adherence to the Mediterranean diet, and compliance with national dietary guidelines. Results: Increased UPF consumption was not inherently associated with healthy or unhealthy specific food groups, although children and adolescents who consumed UPF daily were less likely to exhibit high overall diet quality and adherence to the Mediterranean diet. In all five countries, greater UPF intake was associated with poorer compliance with dietary recommendations concerning fats, sweets, meat, and legumes. Conclusions: Increased UPF consumption among Mediterranean children and adolescents is associated with an unhealthy dietary pattern, possibly marked by a high intake of fats, sweets, and meat, and a low consumption of legumes.

## 1. Introduction

Today’s diet has undergone profound changes compared to the past, with an evolution that has seen a gradual shift away from fresh, seasonal, and local products in favor of increasingly industrialized and processed foods, widely consumed by children and adolescents [1]. This phenomenon of food processing, linked to globalization and technological progress, has radically transformed the way food is produced, preserved, and consumed, becoming a central element in contemporary food production as well as a highly debated topic due to the potential negative effects on human health associated with the consumption of such foods [2]. Children represent the most critical and vulnerable population affected by worsening dietary habits due to their unique developmental needs and long-term health implications [3]. During childhood, nutrition plays a foundational role in physical growth, cognitive development, and immune system function [4]. Poor dietary patterns established early can disrupt normal growth trajectories and increase the risk of obesity, type 2 diabetes, and cardiovascular diseases later in life as well as affect cognitive and neurological abilities [5]. Moreover, children are highly impressionable and susceptible to environmental influences, including the aggressive marketing of unhealthy foods [6]. Their limited ability to make informed dietary choices makes them especially dependent on caregivers, school systems, and public policies for nutritional guidance [7]. In parallel, there has been a progressive move away from the principles of the Mediterranean diet, traditionally considered a healthy and balanced dietary model, with a concomitant shift towards the eating habits typical of the “Western diet” [8,9]. This transition, characterized by a significant increase in the consumption of calorie-dense, nutrient-poor foods is particularly evident among children and young adults [10]. Compared to countries more affected by the Western culture (i.e., the US, Canada, UK, and Australia), Mediterranean countries have been reported to have a generally lower consumption of industrial processed unhealthy foods [11], although current trends are worrying and health of future generations may be shaped by current dietary habits. In this context, it is important to assess the inclusion of industrial foods within the context of current diets in Mediterranean countries.

According to the Nova classification based on the level of transformation of food stuff, ultra-processed foods (UPFs) are defined as industrially manufactured products that contain little or no whole foods and are distinguished by aesthetic alterations and the addition of additives designed to enhance sensory properties [12]. In addition to techniques such as pasteurization, sterilization, and freeze-drying, the use of chemical additives has allowed for the extension of food shelf life, improved convenience, and year-round availability, regardless of seasonality [13]. These processes, while certainly making important changes in terms of food shelf life, may also significantly influence the nutritional quality of foods, often making them rich in sugars, saturated fats, and salt to increase palatability, while leading to the loss of vitamins, minerals, and other essential nutrients [14]. Furthermore, the increasing use of preservatives, colorings, and artificial flavorings raises concerns about human health, with studies highlighting potential long-term effects related to the consumption of UPF, such as an increased risk of obesity, type 2 diabetes, cardiovascular diseases, early onset of obesity and other chronic conditions related to cardio-metabolic disorders [15]. Additionally, there is growing scientific evidence linking the excessive consumption of such foods to cognitive deficits and learning difficulties, negatively affecting brain development during childhood [16].

In addition to having negative health implications, the consumption of UPF is often associated with a significantly lower overall diet quality [17]. This is particularly important especially in children and adolescents, since consuming these foods from childhood can also negatively impact long-term eating habits, contributing to the development of poor dietary behaviors [4]. Recent studies have shown that those who regularly consume large amounts of such foods tend to have an unbalanced nutritional profile, characterized by the insufficient intake of essential micronutrients, such as vitamins and minerals, and an excessive consumption of low-quality macronutrients, such as refined sugars and saturated fats [18]. This relationship goes beyond the simple nutritional content of such specific foods and may also involves the overall eating habits of children and adolescents: those who consume large amounts of UPFs may adopt unhealthy eating behaviors, such as poor variety in food choices and an insufficient consumption of fruits, vegetables, and whole grains, with a preference for ready-to-eat meals or calorie-dense snacks [19].

Evidence on the detrimental effects of UPFs on human health is currently debated [14]. As a large share of daily energy intake from UPF is characterized by nutritionally inadequate products [20], several researchers argue that the negative outcomes associated with higher intake of UPFs are strongly (if not unequivocally) affected by worse nutritional content in the diet (i.e., high energy from fats and sugars). However, it is unclear whether unhealthy UPFs are related to worse overall diet quality. The aim of this study was to investigate the consumption of unhealthy UPF in children and adolescents living in five Mediterranean countries participating in the DELICIOUS project (“Understanding consumer food choices & promotion of healthy and sustainable Mediterranean diet and lifestyle in children and adolescents through behavioral change actions”) and to evaluate its relationship with dietary quality parameters, including their adherence to the Mediterranean diet.

## 2. Materials and Methods

### 2.1. Study Population

The sample analyzed in this study includes parents providing information on dietary and lifestyle habits of children and adolescents (age 6–17 years old) living in five Mediterranean countries (Italy, Spain, Portugal, Egypt, and Lebanon) participating in the DELICIOUS project: a detailed description of the methodology is provided elsewhere [21]. This preliminary survey had the main aim of assessing the status of adherence to the Mediterranean diet and the many factors associated with it among children and adolescents living in the aforementioned Mediterranean countries. Participants were recruited through a consumers database established by one of the study partners. Participants had to meet the criteria of having an internet connection and being parents of children falling into the target age range. According to recent scientific articles published on the same topic and sharing a similar methodology, an ideal sample size of 400 participants per country was considered sufficient to observe significant differences when exploring determinants of the main outcome of interest (i.e., adherence to the Mediterranean diet) [22,23]. The data was collected through an electronic survey with a total of 2011 participants ultimately recruited to participate in the study. All procedures followed the guidelines of the World Medical Association’s Declaration of Helsinki (1989). All study participants provided informed consent prior to involvement in the study.

### 2.2. Data Collection

Data on the background characteristics of children and adolescents, such as sex, age, weight status, and physical activity level was collected. The weight status was categorized based on the children’s and adolescents’ body mass index (BMI), which was calculated following the percentile groups [normal weight (BMI 5th–84th percentile), overweight (BMI 85th–94th percentile), and obese (BMI ≥ 95th percentile)] provided by the Centers for Disease Control and Prevention (CDC) growth charts for children and adolescents aged 2 to 19 years [24]. Physical activity levels were estimated by administering the International Physical Activity Questionnaire—Short Form (IPAQ), a flexible tool which uses information referring to type and intensity physical activity (walking, moderate and vigorous intensity activities) over the past seven days. Physical activity levels were classified as low, moderate, and high according to IPAQ guidelines [25].

### 2.3. Dietary Assessment

Dietary habits were assessed by administering parents with 24 h recalls of their children’s food intake providing response options for each eating occasion and an open blank option to eventually include additional foods. The average intake of food per week was recorded through food frequency questions on major food groups of interest. Specifically concerning unhealthy UPFs, a set of specific questions on 13 food groups typically falling into the Nova group 4 was used to collect information on frequency consumption of fast foods, soft drinks, pastries, candies, etc. The frequency consumption was then categorized as (i) “high consumption” when falling over the upper median frequency, and (ii) “daily consumption” if at least one food group was reported to be consumed on a daily basis.

### 2.4. Diet Quality Measures

Several measures aimed to assess diet quality were used as outcomes of interest in this study. Food-based dietary recommendations were retrieved for each country based on available resources for Italy [26], Portugal [27], Spain [28], Lebanon [29], and Egypt [30]. For those countries with no available guidelines for children and adolescents, dietary recommendations for adults were considered, assuming portion sizes proportional to reference age. To evaluate other quantitative measures of diet quality, the Youth Healthy Eating Index (Y-HEI) [31] and the Mediterranean Diet Quality Index (KIDMED) [32] were applied to the dietary data retrieved. The Y-HEI is a tool used to screen the diet quality of older children and adolescents assessing the consumption of 13 food sources of fats, fiber, sodium, and added sugars including whole-grains, fruit and vegetables, dairy, meat ratio, snack foods, soda and drinks, multivitamin use, margarine and butter, fried foods, visible animal fats, as well as breakfast eating and dinner with family. The scoring system ranges from 0 to 100 (but for the purposes of this study, data on multivitamin intake and visible fats were not assessed, leading to a maximum possible score of 90), with a score over the upper tertile reflecting better diet quality. The KIDMED is composed of 16 questions on a set of food items in line (or misaligned) with the Mediterranean dietary model to which positive or negative scores are assigned for a maximum total possible score of 12, with a score ≥ 7 deemed as having high adherence to the Mediterranean diet.

### 2.5. Statistical Analysis

Categorical variables are presented as frequencies and percentages, with the Chi-square test used to assess differences between groups of UPF consumption. Continuous variables are expressed as means and standard deviations (SDs), with the ANOVA test used to examine differences between groups. Multivariate logistic regression models adjusted for sex, age group, weight status, and physical activity level were applied to calculate odds ratios (ORs) and 95% confidence intervals (CIs) for the association between UPF consumption and diet quality measures. All reported *p*-values were based on two-sided tests and compared to a significance level of 5%. SPSS 21 software (SPSS Inc., Chicago, IL, USA) was used for all statistical calculations.

## 3. Results

The main demographic features of children and adolescents included in the study according to UPF consumption are outlined in Table 1. The results indicate the absence of significant differences in sex in more frequent and daily UPF consumption. Significant differences, however, were observed concerning the weight status, with a higher proportion of overweight and obese children/adolescents among those consuming more daily and total UPF was observed. Also, among high UPF consumption there was a higher proportion of more physically active adolescents (Table 1).

Table 2 shows the frequency distribution of major food groups by level of consumption and daily consumption of UPFs. The findings revealed that there was a significantly higher proportion of children and adolescents eating more cereals, meat, nuts, and sweets among high and daily UPF consumers; also, dairy intake was higher in high UPF consumers. In contrast, a higher proportion of intermediate fruit and legumes intake was registered among high and daily UPF consumers. No differences by level of UPF consumption in the distribution of intake of vegetables and whole grains was found. Country-specific data partially reflected the pooled analyses, with preference for higher consumption of meat and sweets among high UPF consumers across countries (Supplementary Table S1). However, some inconsistent findings occurred for frequency of fruit intake, which resulted higher in high UPF consumers in Spain (Supplementary Table S1).

Table 3 illustrates the association between the consumption of unhealthy UPFs and measures of diet quality, such as Y-HEI and the KIDMED. After adjusting for potential confounding factors (including adjusted for sex, age group, weight status, and physical activity level), the results indicate that children and adolescents with a high daily and total consumption of unhealthy UPFs exhibited significantly lower scores on the Y-HEI (OR = 0.47, 95% CI: 0.38, 0.59 and OR = 0.40, 95% CI: 0.29, 0.55, respectively) and those consuming UPFs daily were less likely to have high adherence to the Mediterranean diet (OR = 0.63, 95% CI: 0.45, 0.88). Subgroup analyses by country confirmed significant inverse association between high UPF consumption and diet quality in Egypt, Italy, and Portugal and null findings for Spain and Lebanon, with the latter showing incongruent associations with higher adherence to the Mediterranean diet (Supplementary Table S2).

**Figure 1:**
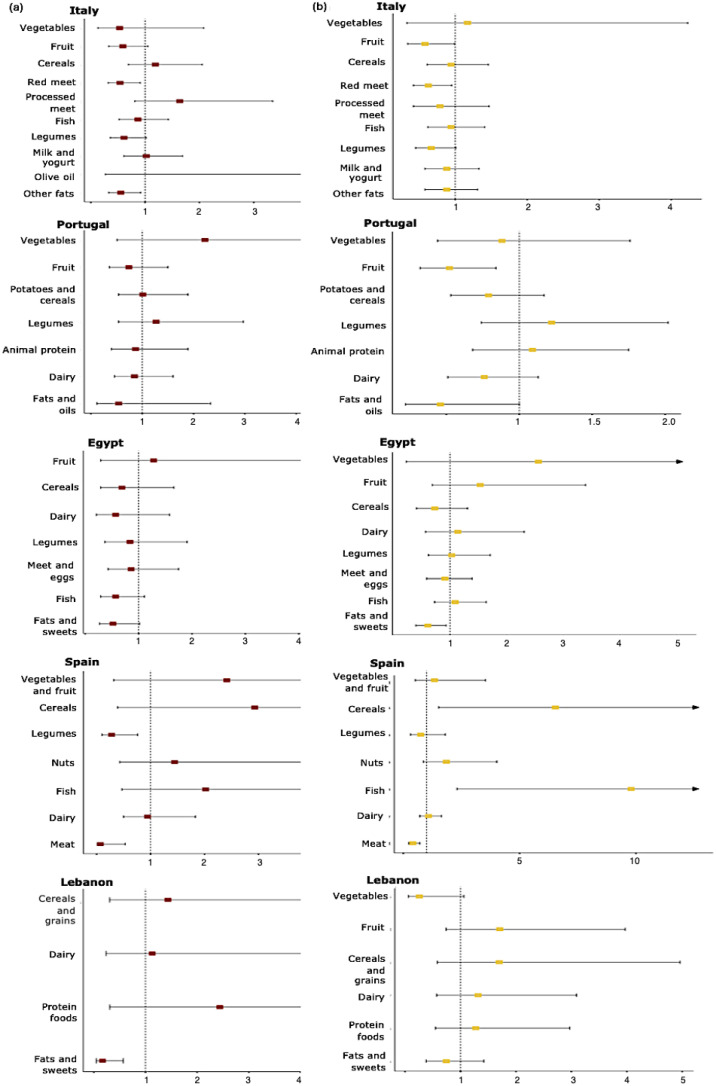
Association between (**a**) daily UPF and (**b**) high UPF consumption and adequate adherence to individual country-specific dietary recommendations. Squares denote odds ratios (ORs) and lines denote 95% confidence intervals.

Figure 1 illustrates the graphical representation of the probability (ORs and 95% CI) of meeting country-specific dietary recommendations for daily and high unhealthy UPF consumption. A lower probability of adhering to dietary guidelines on fats in Italy, Egypt, Spain, and Lebanon was associated with daily UPF consumption; also lower odds of meeting the guidelines on red meat (in Italy and Spain) and legumes (in Spain) were found (Figure 1). Individuals reporting high UPF consumption were less likely to adhere to national recommendations on fruit (in Italy and Portugal), fats, oils, and sweets (in Portugal and Egypt), and meat (in Spain); in contrast, they had a higher probability for adhering to the Spanish recommendations on fish (Figure 1).

## 4. Discussion

This study aimed to investigate the association between the consumption of unhealthy UPFs and overall diet quality, including adherence to the Mediterranean diet, in children and adolescents from five Mediterranean countries. In particular, the study demonstrates how the consumption of unhealthy UPFs is closely linked to poorer overall diet quality. These findings are supported by other studies that have demonstrated a correlation between the consumption of UPFs and poor nutritional quality of the diet, both in children and adults, also highlighting how children’s food choices are influenced by those of their parents [33]. In addition, the present study aimed to investigate the association between the consumption of unhealthy UPFs among children and adolescents in the Mediterranean region and their adherence to the Mediterranean dietary pattern. The results indicated that participants with higher UPF consumption exhibited lower adherence to the Mediterranean diet, although not consistently across countries. Other studies have also highlighted the poor adherence to the Mediterranean diet and the increased consumption of UPFs or junk food in children and adolescents living in the Mediterranean area [34,35,36,37]. This suggests a significant correlation between the high intake of UPFs and the decline in following a traditional, balanced dietary model such as the Mediterranean diet [38], which is characterized by a high consumption of plant-based foods, seasonal and minimally processed ingredients, olive oil as the main fat, moderate dairy and fish intake, limited red meat [39]. The current literature shows that individuals with higher UPF intake tend to favor energy-dense, nutrient-poor foods that are widely promoted and easily accessible, often at the expense of healthier, minimally processed alternatives [40]. Children and adolescents who consume more UPFs often favor unhealthy, easily accessible foods, frequently promoted through marketing strategies such as junk food, over more nutritious and healthier options [41].

Healthy lifestyles have been shown to be associated with higher adherence to the Mediterranean diet and overall higher diet quality [42,43]. Contrary to the current literature, the results from this study showed that more frequent consumption of unhealthy UPF was related to higher physical activity levels. One can hypothesize that in the present sample more active young people would be more likely to eat energy-rich foods eventually due to increased hunger from higher physical activity and not because of an unhealthy lifestyle. However, this hypothesis needs to be further confirmed, since most scientific literature agrees that unhealthy lifestyles tend to cluster [44].

The analysis of dietary habits among the children and adolescents involved in the study revealed that those who consume a high amount of unhealthy UPFs on a daily basis also tend to consume greater quantities of cereals, meat, nuts, and sweets. These findings align with the results related to the daily consumption of UPFs, suggesting that the consumption of unhealthy UPFs (i.e., sweets) may cluster with other food groups unmeasured as ultra-processed (i.e., cereals, meat, and nuts). For instance, some of such foods may be consumed in packaged and industrial forms. To date, a significant portion of the meat consumed by children and adolescents is represented by UPF products, such as dried meats, sausages, processed cold cuts, and pre-packaged meats [45]. These foods are not only easier to prepare and perceived as more flavorful, but they also are more likely to be consumed in fast foods and outside the home [46]. Cereals are commonly consumed as breakfast products, often transformed into unhealthy alternative products [47]. Similarly, nuts can be consumed within the context of snacks and sweets, which can be processed and packaged products containing additives (such as sugars or preservatives to prolong shelf life and enhance taste) [48]. While nuts are generally considered a healthy source of nutrients, industrial versions may have an altered nutritional profile due to the addition of unhealthy ingredients [49]. Sweets are often consumed in industrial forms, including biscuits, cakes, pastries, and candies [50], which are generally enriched with saturated fats, refined sugars, and preservatives, which increase their calorie content and reduce their overall nutritional value [51]. Conversely, the consumption of other food groups, such as vegetables and whole grains was not related with the intake of unhealthy UPFs. Moreover, country-specific data revealed that higher UPF consumption was associated even with higher intake of fruit or vegetables in certain countries (i.e., Spain and Lebanon). We can hypothesize that these food groups are mostly unrelated to the preference for UPF since their most common forms are generally unprocessed and their average consumption is already generally low. In addition, while the other food groups explored may be affected by convenience and ease of access (i.e., sweets do not need to be cooked), vegetables and whole grains are more likely to depend on flavor and cooking skills, which in turn may rely on parents’ wills. Also, fruits may accompany snacking in highly physically active young people, which also resulted in higher UPF consumption: again, we can hypothesize that more physically active young people are simply more hungry, and increase the intake of several food groups, especially those easy to consume (i.e., UPFs but also fruits).

The results presented in this study suggest that the consumption of UPFs is not limited to traditionally unhealthy items but also includes foods that, if consumed fresh or in less processed forms, could offer nutritional benefits [52]. The high consumption of these packaged foods may be influenced by convenience, marketing, and the taste preferences of younger populations [6]. Additionally, it could also be related to socio-economic factors, such as the availability of fresh foods and limited time to prepare balanced meals [53]. Finally, the cultural and social context may play an important role [54]. In many Mediterranean regions, the availability and accessibility of fresh foods vary considerably [55]. Families living in urban areas, for example, may have limited access to fresh fruit and bulk legumes, preferring packaged alternatives for reasons of practicality or preservation [56]. Similarly, marketing and advertising strategies for UPFs, often targeted at children, could contribute to an increased consumption of these products, indirectly influencing how they are consumed [57].

To the best of our knowledge, this study stands out for its ability to analyze the association between the consumption of unhealthy UPFs and overall diet quality, including adherence to the Mediterranean diet, in children and adolescents from five Mediterranean countries, using a standardized methodology. However, the conclusions of this study should be interpreted in light of certain limitations. Firstly, the cross-sectional design of the study does not allow for the establishment of causal relationships. Additionally, reporting bias may be present due to the questionnaires completed by parents, who reported the dietary frequencies and eating habits of their children. Moreover, both 24 h recall and food frequency questionnaires suffer from recall and social desirability bias; also, these instruments tend to under and overestimate dietary intakes, respectively. However, they still represent the gold standard to be used in nutritional epidemiology and no better instruments with such flexible usability are currently available. Finally, the estimation of UPF consumption was only limited to unhealthy foods (mostly junk foods), which are not comprehensive of all food groups representing the totality of UPFs included in the diet.

## 5. Conclusions

In conclusion, the consumption of unhealthy UPFs among children and adolescents residing in the Mediterranean region is significantly associated with poor diet quality and low adherence to the Mediterranean dietary pattern. This finding highlights an inverse relationship between UPF intake and overall diet quality. The data representation provides a detailed analysis of the connections between participants’ eating habits and diet quality parameters, offering a comprehensive perspective on the implications of UPF consumption within the context of a Mediterranean dietary model. To effectively reduce unhealthy UPF consumption, a comprehensive, multi-sectoral strategy is required, targeting the structural drivers of dietary behaviors. A holistic approach that incorporates nutritional education, awareness about the risks of UPFs, and practical strategies is crucial to encourage balanced diets and healthy lifestyle habits among children and adolescents. Collaboration between parents, educators, and healthcare professionals is essential to foster a supportive environment that promotes nutritious food choices and enhances the overall well-being of future generations. Regulatory measures should be implemented to restrict the marketing of UPFs, particularly to children, who are especially susceptible to persuasive advertising tactics that shape long-term consumption patterns. School environments must be safeguarded through stringent nutritional standards for meals and canteens, ensuring access to minimally processed, nutrient-dense foods that support healthy development. Simultaneously, governments and public health institutions must hold the food industry accountable by enforcing transparent front-of-pack labeling, reformulation targets to reduce sugar, salt, and saturated fat in processed products, and fiscal policies such as the taxation of high-risk food categories. These actions, underpinned by public education and community engagement, would aim not only to reduce exposure to unhealthy products but also to create an enabling environment that supports informed choices and promotes equitable access to nutritious foods.

## Figures and Tables

**Table 1:** The demographic characteristics of parents and children/adolescents participating in the study according to the level of unhealthy UPF consumption (n = 2011).

		Consumption of UPF			Daily Consumption of UPF		
	Total (n = 2011)	Low (n = 880)	High (n = 1131)	*p*-Value ^a^	No (n = 212)	Yes (n = 1799)	*p*-Value ^a^
**Age groups, n (%)**				0.040			0.122
Children	1047 (52.1)	481 (54.7)	566 (50.0)		121 (57.1)	926 (51.5)	
Adolescents	964 (47.9)	399 (45.3)	565 (50.0)		91 (42.9)	873 (48.5)	
**Sex, n (%)**				0.064			0.057
Male	995 (49.5)	456 (51.8)	539 (47.7)		118 (55.7)	877 (48.7)	
Female	1016 (50.5)	424 (48.2)	592 (52.3)		94 (44.3)	922 (51.3)	
**Weight status, n (%)**				<0.001			0.010
Normal weight	1087 (68.7)	551(74.4)	536 (63.0)		130 (78.8)	957 (67.5)	
Overweight	263 (16.6)	107 (14.6)	156 (18.3)		21 (12.7)	242 (17.1)	
Obese	232 (14.7)	73 (10.0)	159 (18.7)		14 (8.5)	218 (15.4)	
**Physical activity level, n (%)**				0.047			0.210
Low	1017 (50.6)	470 (53.4)	547 (48.4)		110 (51.9)	907 (50.4)	
Medium	461 (22.9)	182 (20.7)	279 (24.7)		39 (18.4)	422 (23.5)	
High	533 (26.5)	228 (25.9)	305 (27.0)		63 (29.7)	470 (26.1)	

^a^*p*-values refer to Chi-square tests.

**Table 2:** Eating behaviors of children and adolescents according to the level of unhealthy UPF consumption.

	Consumption of UPF			Daily Consumption of UPF		
	Low	High	*p*-Value ^a^	Low	Hight	*p*-Value ^a^
**Vegetables, n (%)**			0.268			0.416
Never	57 (6.5)	68 (6.0)		11 (5.2)	114 (6.3)	
1–2 portion/d	732 (83.2)	920 (81.3)		171 (80.7)	1481 (82.3)	
≥3 portion/d	91 (10.3)	143 (12.6)		30 (14.2)	204 (11.3)	
**Fruit, n (%)**			0.026			0.001
Never	52 (5.9)	41 (3.6)		19 (9.0)	74 (4.1)	
1–2 portion/d	658 (74.8)	890 (78.7)		144 (67.9)	1404 (78.0)	
≥3 portion/d	170 (19.3)	200 (17.7)		49 (23.1)	321 (17.8)	
**Cereals, n (%)**			<0.001			<0.001
Never	61 (6.9)	45 (4.0)		21 (9.9)	85 (4.7)	
1–2 portion/d	786 (89.3)	915 (80.9)		181 (85.4)	1520 (84.5)	
≥3 portion/d	33 (3.8)	171 (15.1)		10 (4.7)	194 (10.8)	
**Dairy, n (%)**			<0.001			0.406
Never	188 (21.4)	216 (19.1)		50 (23.6)	354 (19.7)	
1–2 portion/d	534 (60.7)	627 (55.4)		117 (55.2)	1044 (58)	
≥3 portion/d	158 (18.0)	288 (25.5)		45 (21.2)	401 (22.3)	
**Meat, n (%)**			0.009			<0.001
Never	80 (9.1)	63 (5.6)		34 (16.0)	109 (6.1)	
1–2 portion/w	442 (50.2)	599 (53)		118 (55.7)	923 (51.3)	
≥3 portion/w	358 (40.7)	469 (41.5)		60 (28.3)	767 (42.6)	
**Legumes, n (%)**			0.101			0.032
Never	44 (5.0)	57 (5.0)		9 (4.2)	92 (5.1)	
1–2 portion/w	590 (67.0)	805 (71.2)		133 (62.7)	1262 (70.2)	
≥3 portion/w	246 (28.0)	269 (23.8)		70 (33.0)	445 (24.7)	
**Fish, n (%)**			<0.001			<0.001
Never	160 (18.2)	125 (11.1)		53 (25.0)	232 (12.9)	
1–2 portion/w	556 (63.2)	814 (72.0)		116 (54.7)	1254 (69.7)	
≥3 portion/w	164 (18.6)	192 (17.0)		43 (20.3)	313 (17.4)	
**Nuts, n (%)**			<0.001			<0.001
Never	437 (49.7)	316 (27.9)		107 (50.5)	646 (35.9)	
1–2 portion/w	374 (42.5)	678 (59.9)		86 (40.6)	966 (53.7)	
≥3 portion/w	69 (7.8)	137 (12.1)		19 (9.0)	187 (10.4)	
**Whole grains, n (%)**			0.334			0.506
Never	244 (27.7)	323 (28.6)		59 (27.8)	508 (28.2)	
1–2 portion/w	343 (39.0)	466 (41.2)		79 (37.3)	730 (40.6)	
≥3 portion/w	293 (33.3)	342 (30.2)		74 (34.9)	561 (31.2)	
**Sweets, n (%)**			<0.001			<0.001
Never	113 (12.8)	47 (4.2)		49 (23.1)	111 (6.2)	
1–2 portion/w	468 (43.2)	447 (39.5)		131 (61.8)	784 (43.6)	
≥3 portion/w	299 (34.0)	637 (56.3)		32 (15.1)	904 (50.3)	

^a^*p*-values refer to Chi-square tests.

**Table 3:** Association between diet quality, Mediterranean diet of children and adolescents, and consumption of unhealthy UPFs (n = 2011).

	Y-HEI			Mediterranean Diet		
	Low	High	OR (95% CI) ^a^	Low	High	OR (95% CI) ^a^
**High UPF intake, n (%)**	1281 (71.2)	518 (28.8)		1304 (72.5)	495 (27.5)	
Unadjusted			0.47 (0.39, 0.57)			1.17 (0.96, 1.42)
Adjusted *			0.47 (0.38, 0.59)			1.04 (0.83, 1.30)
**Daily UPF intake, n (%)**	862 (76.2)	269 (23.8)		798 (70.6)	333 (29.4)	
Unadjusted			0.44 (0.33, 0.58)			0.77 (0.57, 1.04)
Adjusted *			0.40 (0.29, 0.55)			0.63 (0.45, 0.88)

^a^ OR measures were assessed through logistic regression tests. * adjusted for sex, age group, weight status, and physical activity level.

## Data Availability

The original contributions presented in the study are included in the article/Supplementary Material, further inquiries can be directed to the corresponding author.

## Supplementary Materials

The following supporting information can be downloaded at: https://www.mdpi.com/article/10.3390/foods14152648/s1, Table S1. Eating behaviors of children and adolescents according to the level of unhealthy UPF consumption, by country; Table S2. Eating Association between diet quality and Mediterranean Diet of children and adolescents and consumption of unhealthy UPFs.

## Abbreviations

The following abbreviations are used in this manuscript:

UPF	Ultra-processed food
BMI	Body Mass Index
CDC	Centers for Disease Control and Prevention
IPAQ	International Physical Activity Questionnaire
Y-HEI	Youth Healthy Eating Index
KIDMED	Mediterranean Diet Quality Index
SD	Standard Deviation
OD	Odds Ratio
CI	Confidence Interval
