# Supplementary material for: Unhealthy Ultra-Processed Food, Diet Quality and Adherence to the Mediterranean Diet in Children and Adolescents: The DELICIOUS Project
Source: Foods. 2025 Jul 28;14(15):2648. doi: 10.3390/foods14152648 (PMC12345705; doi:10.3390/foods14152648)
Supplement: Supplementary file 1 [file foods-14-02648-s001.zip › foods-3642845-supplementary.pdf]

Supplementary Table S1. Eating behaviors of children and adolescents according to the level of unhealthy UPF consumption, by country.

	Spain (n = 411)		Egypt (n = 400)		Italy (n = 402)		Lebanon (n = 398)		Portugal (n = 400)	
	Consumption of UPF		Consumption of UPF		Consumption of UPF		Consumption of UPF		Consumption of UPF	
	Low	High	Low	Hight	Low	Hight	Low	Hight	Low	Hight
**Vegetables, n (%)**										
Never	11 (7.7)	11 (4.1)	16 (7.1)	14 (8.0)	19 (7.4)	18 12.3)	1 (2.1)	9 (2.6)	10 (4.7)	16 (8.5)
1-2 portion/d	119 (83.8)	217 (80.7)	186 (83.0)	143 (81.3)	204 (79.7)	110 (75.3)	45 (95.7)	284 (80.9)	178 (84.4)	166 (87.8)
≥3 portion/d	12 (8.5)	41 (15.2)	22 (9.8)	19 (10.8)	33 (12.9)	18 (12.3)	1 (2.1)	58 (16.5)*	23 (10.9)	7 (3.7)*
**Fruit, n (%)**										
Never	24 (16.9)	10 (3.7)	11 (4.9)	8 (4.5)	13 (5.1)	13 (8.9)	1 (2.1)	2 (0.6)	3 (1.4)	8 (4.2)
1-2 portion/d	104 (73.2)	215 (79.9)	174 (77.7)	142 (80.7)	188 (73.4)	108 (74.0)	40 (85.1)	279 (79.5)	152 (72.0)	146 (77.2)
≥3 portion/d	14 (9.9)	44 (16.4)**	39 (17.4)	26 (14.8)	55 (21.5)	25 (17.1)	6 (12.8)	70 (19.9)	56 (26.5)	35 (18.5)*
**Cereals, n (%)**										
Never	20	18	5	2	30	13	3	12	3	0

	(14.1)	(6.7)	(2.2)	(1.1)	(11.7)	(8.9)	(6.4)	(3.4)	(1.4)	(0.0)
1-2 portion/d	112 (78.9)	201 (74.7)	214 (95.5)	167 (94.9)	218 (85.2)	129 (88.4)	42 (89.4)	239 (68.1)	200 (94.8)	179 (94.7)
≥3 portion/d	10 (7.0)	50 (18.6)**	5 (2.2)	7 (4.0)	8 (3.1)	4 (2.7)	2 (4.3)	100 (28.5)**	8 (3.8)	10 (5.3)
**Dairy, n (%)**										
Never	46 (32.4)	66 (24.5)	59 (26.3)	35 (19.9)	40 (15.6)	22 (15.1)	7 (14.9)	49 (14.0)	36 (17.1)	44 (23.3)
1-2 portion/d	76 (53.5)	134 (49.8)	147 (65.6)	125 (71.0)	162 (63.3)	99 (67.8)	28 (59.6)	153 (43.6)	121 (57.3)	116 (61.4)
≥3 portion/d	20 (14.1)	69 (25.7)*	18 (8.0)	16 (9.1)	54 (21.1)	25 (17.1	12 (25.5)	149 (42.5)	54 (25.6)	29 (15.3)*
**Meat, n (%)**										
Never	42 (29.6)	46 (17.1)	12 (5.4)	4 (2.3)	14 (5.5)	3 (2.1)	2 (4.3)	5 (1.4)	10 (4.7)	5 (2.6)
1-2 portion/w	81 (57.0)	149 (55.4)	146 (65.2)	113 (64.2)	133 (52.0)	64 (43.8)	29 (61.7)	217 (61.8)	53 (25.1)	56 (29.6)
≥3 portion/w	19 (13.4)	74 (27.5)**	66 (29.5)	59 (33.5)	109 (42.6)	79 (54.1)*	16 (34.0)	129 (36.8)	148 (70.1)	128 (67.7)
**Legumes, n (%)**										

Never	6 (4.2)	20 (7.4)	14 (6.3)	10 (5.7)	12 (4.7)	12 (8.2)	1 (2.1)	8 (2.3)	11 (5.2)	7 (3.7)
1-2 portion/w	110 (77.5)	186 (69.1)	101 (45.1)	99 (56.3)	203 (79.3)	110 (75.3)	38 (80.9)	285 (81.2)	138 (65.4)	125 (66.1)
≥3 portion/w	26 (18.3)	63 (23.4)	109 (48.7)	67 (38.1)	41 (16.0)	24 (16.4)	8 (17.0)	58 (16.5)	62 (29.4)	57 (30.2)
**Fish, n (%)**										
Never	96 (67.6)	82 (30.5)	31 (13.8)	18 (10.2)	19 (7.4)	12 (8.2)	3 (6.4)	7 (2.0)	11 (5.2)	6 (3.2)
1-2 portion/w	44 (31.0)	154 (57.2)	173 (77.2)	137 (77.8)	190 (74.2)	118 (80.8)	42 (89.4)	288 (82.1)	107 (50.7)	117 (61.9)
≥3 portion/w	2 (1.4)	33 (12.3)**	20 (8.9)	21 (11.9)	47 (18.4)	16 (11.0)	2 (4.3)	56 (16.0)*	93 (44.1)	66 (34.9)
**Nuts, n (%)**										
Never	97 (68.3)	70 (26.0)	136 (60.7)	81 (46.0)	105 (41.0)	46 (31.5)	8 (17.0)	37 (10.5)	91 (43.1)	82 (43.4)
1-2 portion/w	36 (25.4)	169 (62.8)	75 (33.5)	80 (45.5)	129 (50.4)	86 (58.9)	36 (76.6)	258 (73.5)	98 (46.4)	85 (45.0)
≥3 portion/w	9 (6.3)	30 (11.2)**	13 (5.8)	15 (8.5)*	22 (8.6)	14 (9.6)	3 (6.4)	56 (16.0)	22 (10.4)	22 (11.6)
**Whole grains, n (%)**										

Never	75 (52.8)	106 (39.4)	67 (29.9)	44 (25.0)	62 (24.2)	42 (28.8)	10 (21.3)	107 (30.5)	30 (14.2)	24 (12.7)
1-2 portion/w	50 (35.2)	117 (43.5)	91 (40.6)	71 (40.3)	94 (36.7)	55 (37.7)	28 (59.6)	155 (44.2)	80 (37.9)	68 (36.0)
≥3 portion/w	17 (12.0)	46 17.1)*	66 (29.5)	61 (34.7)	100 (39.1)	49 (33.6)	9 (19.1)	89 (25.4)	101 (47.9)	97 (51.3)
**Sweets, n (%)**										
Never	12 (8.5)	6 (2.2)	14 (6.3)	3 (1.7)	63 (24.6)	16 (11.0)	4 (8.5)	10 (2.8)	20 (9.5)	12 (6.3)
1-2 portion/w	77 (54.2)	113 (42.0)	75 (33.5)	47 (26.7)	145 (56.6)	60 (41.1)	36 (76.6)	141 (40.2)	135 (64.0)	86 (45.5)
≥3 portion/w	53 (37.3)	150 (55.8)**	135 (60.3)	126 (71.6)*	48 (18.8)	70 (47.9)**	7 (14.9)	200 (57.0)**	56 (26.5)	91 (48.1)**

*denotes p-values <0.05 by Chi-square tests.
**denotes p-values <0.001 by Chi-square tests.

Supplementary Table S2. Eating Association between diet quality and Mediterranean Diet of children and adolescents and consumption of unhealthy UPFs (n = 2,011).

	**Y-HEI, OR (95% CI)**[a]	**Mediterranean diet, OR (95% CI)**[a]
**High UPF intake**		
Spain	0.72 (0.44, 1.18)	3.73 (1.94, 7.17)
Egypt	0.47 (0.29, 0.76)	0.86 (0.53, 1.38)
Italy	0.54 (0.36, 0.82)	0.61 (0.38, 0.97)
Lebanon	0.53 (0.28, 1.01)	7.30 (2.56, 20.80)
Portugal	0.49 (0.32, 0.74)	0.62 (0.41, 0.95)

[a]OR measures were assessed through logistic regression tests. All analyses are adjusted for sex, age group, weight status, and physical activity level.
